# Supplementary material for: Status of Glucocorticoid-Induced Osteoporosis Preventive Care in Korea: A Retrospective Cohort Study on the Korean National Health Insurance Service Database
Source: Medicina (Kaunas). 2022 Feb 21;58(2):324. doi: 10.3390/medicina58020324 (PMC8879589; doi:10.3390/medicina58020324)
Supplement: Supplementary file 1 [file medicina-58-00324-s001.zip › medicina-1548747-supplementary.pdf]

Supplementary Table S1. Comparisons of clinical characteristics according to bone mineral density test

	No BMD test (n=36,591)	BMD test (n=542)	p
Age			
<40 years, n (%)	10,291 (28.1)	16 (3)	<0.001
40~49 years, n (%)	7,765 (21.2)	53 (9.8)	
50~59 years, n (%)	8,678 (23.7)	120 (22.1)	
60~69 years, n (%)	5,692 (15.6)	147 (27.1)	
≥ 70 years, n (%)	4,165 (11.4)	206 (38)	
Age, years, mean ± SD	49.9 ± 15.3	64 ± 12	<0.001
Female, n (%)	18,043 (49.3)	433 (79.9)	<0.001
Residence			
Urban, n (%)	33,443 (91.4)	456 (84.1)	<0.001
Rural, n (%)	3,148 (8.6)	86 (15.9)	
Institution			
Tertiary/general hospital, n (%)	5,720 (15.6)	141 (26)	<0.001
Primary care clinic/nursing hospital, n (%)	30,871 (84.4)	401 (74)	
Initial GCs prescriber specialty			
Rheumatologist, n (%)	392 (1.1)	23 (4.2)	<0.001
Non-rheumatologist internist, n (%)	11,428 (31.2)	180 (33.2)	
Others, n (%)	24,771 (67.7)	339 (62.5)	
GCs requiring conditions			
Systemic autoimmune diseases, n (%)	1,206 (3.3)	68 (12.5)	<0.001
Chronic pulmonary diseases, n (%)	3,099 (8.5)	72 (13.3)	
Others, n (%)	32,286 (88.2)	402 (74.2)	
Cumulative GCs dose*, mg, mean ± SD	204.4 ± 151	268.4 ± 170.8	<0.001
Comorbidities			
Hyperparathyroidism, n (%)	8 (0)	0 (0)	0.736

Hyperthyroidism, n (%)	563 (1.5)	16 (3)	0.008
Hypothyroidism, n (%)	907 (2.5)	22 (4.1)	0.019
Malignancy, n (%)	1,096 (3)	31 (5.7)	<0.001

* Cumulative GCs dose in the first 90 days after the initiation of GCs therapy

BMD bone mineral density, SD standard deviation, GCs glucocorticoids.

Supplementary Table S2. Comparisons of clinical characteristics according to the use of calcium and/or vitamin D.

	No calcium/vitamin D (n=36,522)	Calcium/vitamin D (n=611)	p
Age			
<40 years, n (%)	10,226 (28)	81 (13.3)	0.001
40~49 years, n (%)	7,706 (21.1)	112 (18.3)	
50~59 years, n (%)	8,607 (23.6)	191 (31.3)	
60~69 years, n (%)	5,726 (15.7)	113 (18.5)	
≥ 70 years, n (%)	4,257 (11.7)	114 (18.7)	
Age, years, mean ± SD	48.7 ± 15.3	55.6 ± 14.2	<0.001
Female, n (%)	18,081 (49.5)	395 (64.6)	<0.001
Residence			
Urban, n (%)	33,346 (91.3)	553 (90.5)	0.489
Rural, n (%)	3,176 (8.7)	58 (9.5)	
Institution			
Tertiary/general hospital, n (%)	5,685 (15.6)	176 (28.8)	<0.001
Primary care clinic/nursing hospital, n (%)	30,837 (84.4)	435 (71.2)	
Initial GCs prescriber specialty			
Rheumatologist, n (%)	395 (1.1)	20 (3.3)	<0.001
Non-rheumatologist internist, n (%)	3,112 (8.5)	59 (9.7)	
Others, n (%)	32,314 (88.2)	474 (77.6)	
GCs requiring conditions			
Systemic autoimmune diseases, n (%)	1,196 (3.3)	78 (12.8)	<0.001
Chronic pulmonary diseases, n (%)	3,112 (8.5)	59 (9.7)	
Others, n (%)	32,214	474 (77.6)	
Cumulative GCs dose*, mg, mean ± SD	204 ± 151.1	196.4 ± 168.3	0.272

Comorbidities			
Hyperparathyroidism, n (%)	8 (0)	0 (0)	0.715
Hyperthyroidism, n (%)	560 (1.5)	19 (3.1)	0.002
Hypothyroidism, n (%)	904 (2.5)	25 (4.1)	0.011
Malignancy, n (%)	1,081 (3)	46 (7.5)	<0.001

* Cumulative GCs dose in the first 90 days after the initiation of GCs therapy

SD standard deviation, GCs glucocorticoids.

Supplementary Table S3. Comparisons of clinical characteristics according to the use of osteoporosis medications

	No OP medications (n=36,526)	OP medications (n=607)	p
Age			
<40 years, n (%)	10,271 (28.1)	36 (5.9)	<0.001
40~49 years, n (%)	7,721 (21.1)	97 (16)	
50~59 years, n (%)	8,631 (23.6)	167 (27.5)	
60~69 years, n (%)	5,694 (15.6)	145 (23.9)	
≥ 70 years, n (%)	4,209 (11.5)	162 (26.7)	
Age, years, mean ± SD	49.7 ± 15.3	59.7 ± 13.1	<0.001
Female, n (%)	17,951 (49.1)	525 (86.5)	<0.001
Residence			
Urban, n (%)	33,357 (91.3)	542 (89.3)	<0.001
Rural, n (%)	3,169 (8.7)	65 (10.7)	
Institution			
Tertiary/general hospital, n (%)	5,729 (15.7)	132 (21.7)	<0.001
Primary care clinic/nursing hospital, n (%)	30,797 (84.3)	475 (78.3)	
Initial GCs prescriber specialty			
Rheumatologist, n (%)	406 (1.1)	9 (1.5)	0.538
Non-rheumatologist internist, n (%)	11,411 (31.2)	197 (31.2)	
Others, n (%)	24,709 (67.6)	401 (66.1)	
GCs requiring conditions			
Systemic autoimmune diseases, n (%)	1,220 (3.3)	54 (8.9)	<0.001
Chronic pulmonary diseases, n (%)	3,117 (8.5)	54 (8.9)	
Others, n (%)	32,189 (88.1)	499 (82.2)	
Cumulative GCs dose*, mg, mean ± SD	203.9 ± 150.1	198.8 ± 212.4	<0.001
Comorbidities			
Hyperparathyroidism, n (%)	8 (0)	0 (0)	0.715

Hyperthyroidism, n (%)	566 (1.5)	13 (2.1)	0.243
Hypothyroidism, n (%)	904 (2.5)	25 (4.1)	0.01
Malignancy, n (%)	1,106 (3)	21 (3.5)	0.539

* Cumulative GCs dose in the first 90 days after the initiation of GCs therapy

OP osteoporosis, SD standard deviation, GCs glucocorticoids.

Supplementary Table S4. Associated factors for the bone mineral density test

	Crude OR (95% CI)	p	Adjusted OR (95% CI)	p
Age				
<40 years	1 (ref.)		1 (ref.)	
40~49 years	4.39 (2.5-7.68)	<0.001	4.23 (2.42-7.42)	<0.001
50~59 years	8.89 (5.28-15)	<0.001	8.94 (5.29-15.11)	<0.001
60~69 years	16.61 (9.91-27.85)	<0.001	18.93 (11.25-31.85)	<0.001
≥ 70 years	31.81 (19.1-52.97)	<0.001	34.83 (20.81-58.3)	<0.001
Male	0.25 (0.2-0.3)	<0.001	0.2 (0.16-0.25)	<0.001
Residence				
Urban	1 (ref.)		1 (ref.)	
Rural	2 (1.59-2.53)	<0.001	1.43 (1.13-1.83)	0.004
Institution				
Tertiary/general hospital	1 (ref.)		1 (ref.)	
Primary care clinic/nursing hospital	0.53 (0.43-0.64)	<0.001	0.71 (0.57-0.89)	0.002
Initial GCs prescriber specialty				
Rheumatologist	1 (ref.)		1 (ref.)	
Non-rheumatologist internist	0.27 (0.17-0.42)	<0.001	0.69 (0.4-1.18)	0.172
Others	0.23 (0.15-0.36)	<0.001	0.72 (0.42-1.23)	0.231
Glucocorticoid requiring conditions				
Others	1 (ref.)		1 (ref.)	
Systemic autoimmune diseases	4.53 (3.48-5.89)	<0.001	3.47 (2.62-4.59)	<0.001
Chronic pulmonary diseases	1.87 (1.45-2.4)	<0.001	1.03 (0.78-1.38)	0.05
Cumulative GCs dose*, g	0.1 (0.05-0.22)	<0.001	1.27 (0.83-1.93)	0.387
Comorbidities				
Hyperparathyroidism	N/A	0.973	-	-
Hyperthyroidism	1.95 (1.18-3.22)	0.001	1.66 (1.03-2.66)	0.111
Hypothyroidism	1.67 (1.08-2.56)	0.021	1.13 (0.75-1.72)	0.874
Malignancy	1.97 (1.36-2.84)	<0.001	1.98 (1.43-2.73)	0.056

* Cumulative GCs dose in the first 90 days after the initiation of GCs therapy

OR odds ratio, GCs glucocorticoids

Supplementary Table S5. Associated factors for the use of calcium and/or vitamin D.

	Crude OR (95% CI)	p	Adjusted OR (95% CI)	p
Age				
<40 years	1 (ref.)		1 (ref.)	
40~49 years	1.83 (1.38-2.45)	<0.001	1.77 (1.32-2.36)	0.001
50~59 years	2.8 (2.16-3.64)	<0.001	2.7 (2.07-3.52)	<0.001
60~69 years	2.49 (1.87-3.32)	<0.001	2.54 (1.9-3.4)	<0.001
≥ 70 years	3.38 (2.54-4.5)	<0.001	3.43 (2.55-4.6)	<0.001
Male	0.54 (0.45-0.63)	<0.001	0.5 (0.42-0.59)	<0.001
Residence				
Urban	1 (ref.)		1 (ref.)	
Rural	1.1 (0.84-4.45)	0.489	0.98 (0.75-1.3)	0.924
Institution				
Tertiary/general hospital	1 (ref.)		1 (ref.)	
Primary care clinic/nursing hospital	0.46 (0.38-0.54)	<0.001	0.59 (0.48-0.72)	<0.001
Initial GCs prescriber specialty				
Rheumatologist	1 (ref.)		1 (ref.)	
Non-rheumatologist internist	0.37 (0.23-0.58)	0.001	1.28 (0.75-2.17)	0.364
Others	0.3 (0.19-0.48)	<0.001	1.18 (0.7-1.99)	0.544
Glucocorticoid requiring conditions				
Others	1 (ref.)		1 (ref.)	
Systemic autoimmune diseases	4.43 (3.47-5.67)	<0.001	3.47 (2.62-4.59)	<0.001
Chronic pulmonary diseases	1.29 (0.98-1.69)	0.069	1.03 (0.78-1.38)	0.816
Cumulative GCs dose*, g	0.69 (0.38-1.25)	0.219	1.26 (0.83-1.93)	0.271
Comorbidities				
Hyperparathyroidism	N/A	0.973	-	-
Hyperthyroidism	2.06 (1.3-3.28)	0.002	1.66 (1.03-2.66)	0.036
Hypothyroidism	1.68 (1.12-2.52)	0.012	1.13 (0.75-1.72)	0.551
Malignancy	2.7 (1.97-3.63)	<0.001	1.98 (1.43-2.73)	<0.001

* Cumulative GCs dose in the first 90 days after the initiation of GCs therapy

OR odds ratio, GCs glucocorticoids

Supplementary Table S6. Associated factors for the use of osteoporosis medications.

	Crude OR (95% CI)	p	Adjusted OR (95% CI)	p
Age				
<40 years	1 (ref.)		1 (ref.)	
40~49 years	3.58 (2.44-5.26)	<0.001	3.65 (2.48-5.36)	<0.001
50~59 years	5.52 (3.85-7.92)	<0.001	6.18 (4.29-8.88)	<0.001
60~69 years	7.26 (5.04-10.48)	<0.001	9.52 (6.58-13.78)	<0.001
≥ 70 years	10.98 (7.64-15.78)	<0.001	14.27 (9.88-20.62)	<0.001
Male	0.15 (0.12-0.19)	<0.001	0.13 (0.1-0.16)	<0.001
Residence				
Urban	1 (ref.)		1 (ref.)	
Rural	1.26 (0.97-1.64)	0.079	0.98 (0.76-1.29)	0.922
Institution				
Tertiary/general hospital	1 (ref.)		1 (ref.)	
Primary care clinic/nursing hospital	0.67 (0.55-0.81)	<0.001	0.72 (0.58-0.88)	0.002
Initial GCs prescriber specialty				
Rheumatologist	1 (ref.)		1 (ref.)	
Non-rheumatologist internist	0.78 (0.4-01.53)	0.466	1.89 (0.9-3.95)	0.091
Others	0.73 (0.38-1.43)	0.359	1.93 (0.93-4.02)	0.078
Glucocorticoid requiring conditions				
Others	1 (ref.)		1 (ref.)	
Systemic autoimmune diseases	2.86 (2.14-3.8)	<0.001	2.5 (1.82-3.44)	<0.001
Chronic pulmonary diseases	1.12 (0.84-1.48)	0.442	0.84 (0.62-1.13)	0.236
Cumulative GCs dose*	0.78 (0.45-1.39)	0.41	1.84 (1.23-2.77)	0.003
Comorbidities				
Hyperparathyroidism	N/A	0.971	-	-
Hyperthyroidism	1.39 (0.8-2.43)	0.243	1.04 (0.59-1.82)	0.905
Hypothyroidism	1.69 (1.13-2.54)	0.011	1.08 (0.71-1.63)	0.729
Malignancy	1.15 (0.74-1.78)	0.539	0.85 (0.54-.135)	0.494

* Cumulative GCs dose in the first 90 days after the initiation of GCs therapy

OR odds ratio, GCs glucocorticoids
